# Retraction: Improving boron use efficiency via different application techniques for optimum production of good quality potato (*Solanum tuberosum *L.) in alkaline soil

**DOI:** 10.1371/journal.pone.0272193

**Published:** 2022-08-03

**Authors:** The PLOS ONE Editors

The *PLOS ONE* Editors retract this article [1] because it was identified as one of a series of submissions for which we have concerns about authorship, competing interests, and peer review. We regret that the issues were not addressed prior to the article’s publication.

MT, BA, MAdnan, IAM, SK, SF, MHS, MAli, MM, MAhmad, MR, and SA did not agree with the retraction. MSC and MAES either did not respond directly or could not be reached.
